# Pediatric intraarticular ganglion cyst with extraarticular extension in the knee: A case report

**DOI:** 10.1016/j.ijscr.2025.111044

**Published:** 2025-02-11

**Authors:** Abdulmajeed Alwayil, Raheef Alatassi, Aiman Alshomrani, Khalid Beidas

**Affiliations:** a Department of Orthopedic Surgery, Security Forces Hospital, 3643, Riyadh 11481, Saudi Arabia; b Department of Orthopaedic Surgery, University of Western Ontario, London Health Sciences Center, A9-028, 339 Windermere Road, London N6A 5A5, ON, Canada

**Keywords:** Pediatric, Knee, Intraarticular, Ganglion cyst, Infra patellar, Arthroscopy, Case report

## Abstract

**Introduction:**

In total, there are fewer than ten reported cases of intraarticular ganglion cysts in the pediatric population. Herein, we report a rare case of intraarticular ganglion cyst inside the knee with extraarticular extension in a 5-year-old child.

**Case presentation:**

A 5-year-old boy presented with an anterior knee mass associated with limping, and limited range of motion in the right knee for 1 year. Surgical excision with arthroscopy was performed to treat the intraarticular ganglion cyst with extraarticular extension. At follow-up, the mass had completely detached, and the patient had full range of motion without complaints or recurrence.

**Clinical discussion:**

Ganglion cysts are benign lesions, and are extremely rare in pediatric age groups with fewer than ten cases reported in the literature. We report this case as the first infrapatellar fat pad ganglion cyst with anterolateral extraarticular extension via the lateral reticulum, compared to previous reported cases, which were pure intraarticular cysts the majority of which had an ACL origin.

**Conclusion:**

The clinical manifestation of a rare ganglion cyst in the knee is variable. Magnetic resonance imaging is the cornerstone of diagnosis. Treatment varies according to the cyst location, and requires analysis of the mass, meticulous preoperative planning, and experienced surgeons.

## Highlights

• There are fewer than ten reported cases of intraarticular ganglion cysts in the pediatric population.
• The clinical manifestation of a rare ganglion cyst in the knee is variable. Magnetic resonance imaging is the cornerstone of diagnosis.
• Treatment varies according to the cyst location, and requires analysis of the mass, meticulous preoperative planning, and experienced surgeons.
• The reported case is a rare intraarticular ganglion cyst within the knee with extraarticular extension in a 5-year-old child.
• The ganglion cyst was excised through knee arthroscopy; the skin was incised along the anterolateral portal, which was approximately 1.5 cm in length, to excise the extraarticular extension of the cyst. Then, by utilizing the same incision, the arthroscope was used to fully extract the remnant intraarticular portion of the cyst

## 1 Introduction

Ganglion cysts are benign soft tissue tumors that could arise from several structures including the bursae, tendons, ligaments, subchondral bone, and joint capsules [1,2]. Approximately 70 % of ganglion cysts are discovered at the wrist joint and the hand. However, ganglion cysts of the knee joint are rare [3,4]. Knee intraarticular ganglion cysts were first described in 1942 by Caan during autopsy [5]. To date, they are considered uncommon lesions with an overall incidence of <2 % in adults. However, they are extremely rare in pediatric age groups [[6], [7], [8], [9]]. A few case reports have reported such lesions in pediatric age groups, with distinct features among cases [[8], [9], [10], [11]]. In most reported cases, the ganglion cysts originated or attached to the anterior cruciate ligament (ACL), posterior cruciate ligament (PCL), menisci, and popliteus tendon. Infra-patellar fat pad cysts are rare type of intraarticular ganglion cyst [6,7,12]. In total, there are fewer than ten reported cases of intraarticular ganglion cysts in the pediatric population.

Herein, we report a rare case of intraarticular ganglion cyst inside the knee with extraarticular extension in a 5-year-old child.

## 2 Statement of informed consent

Informed consent for publication of the case was obtained from the parents of the patient.

## 3 Case report

A 5-year-old male patient presented to our hospital after his parents noticed a right knee mass that had appeared 1 year prior. The mass had gradually grown over the previous year with minimal discomfort during knee motion. His parents noted an unsteady gait while he was walking. Furthermore, he had no history of trauma to the right knee or any chronic medical illnesses.

Upon examination, he had an apparent gelatinous and soft mass on the anterior aspect of his right knee that was approximately 1 cm distal to the popliteal fossa and measured approximately 2 × 2 cm. There was no tenderness over the mass. The mass was slightly mobile and increased in size and became prominent with knee flexion (Video 1). However, the child had full range of motion of the knee but with noticeable limping during walking.

**Fig. 1:**
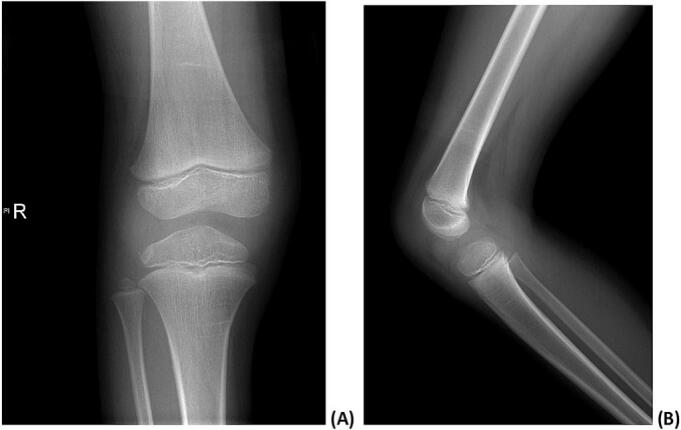
Anteroposterior (A) and lateral (B) radiographs of the right knee that revealed no lesions in the bone or soft tissue.

**Fig. 2:**
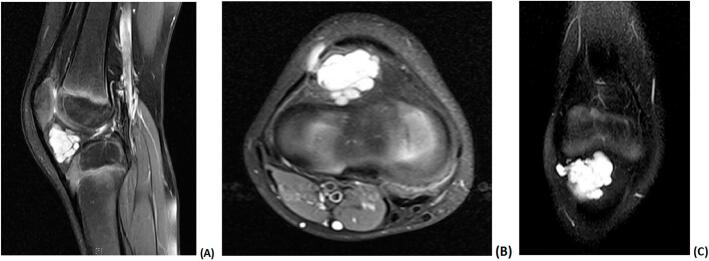
(A), (B) and (C) T1 magnetic resonance imaging of the right knee revealing a hyperintense multilocular cyst suggestive of an intraarticular ganglion cyst in the infrapatellar Hoffa's fat pad with focal extension to the subcutaneous fat via the lateral aspect of the joint capsule and lateral patellar retinaculum.

Neither anteroposterior nor lateral plain radiographs revealed any lesion or abnormalities at the right knee (Fig. 1). Magnetic resonance imaging (MRI) of the right knee revealed a well-defined hyperintense lesion at the infrapatellar Hoffa's fat pad that was multi locular and intraarticular with focal extension to the subcutaneous fat via the lateral aspect of the joint capsule and lateral patellar retinaculum. The underlying bone marrow osseous integrity and signal intensity were preserved. Both the anterior and posterior cruciate ligaments and the menisci were intact. Moreover, other structures in the knee exhibited a normal appearance including the collateral ligaments, patellofemoral ligaments, quadriceps, and patellar tendons. The cyst measured approximately 2.3 × 2 × 1.9 cm, as indicated in Fig. 2. Based on the clinical and radiological findings, intraarticular ganglion cyst was suspected, and the patient was admitted to the hospital for its elective excision.

**Fig. 3:**
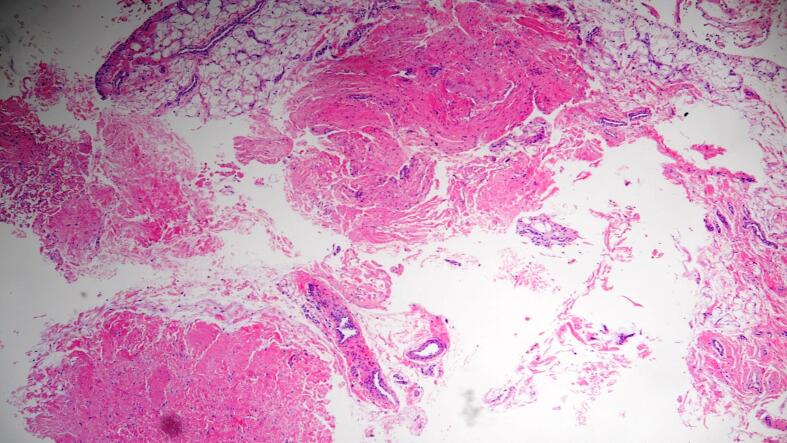
Histopathological slide of the removed tissue revealed a fibrovascular and adipose tissue contents without any epithelial lining which are consistent with ganglion cysts.

During knee arthroscopy, the skin was incised along the anterolateral portal, which was approximately 1.5 cm in length, to excise the extraarticular extension of the cyst. Then, by utilizing the same incision, the arthroscope was used to fully extract the remnant intraarticular portion of the cyst. Aspiration of the cyst revealed a yellow viscous fluid that was sent for histopathological evaluation. Examination of the removed tissue revealed fibrovascular and adipose tissue contents without any epithelial lining. Moreover, it contained synovial cells and macrophages, which are consistent with ganglion cysts as indicated in Fig. 3.

Regarding the postoperative protocol, the patient was advised to weight bear as tolerated on the first day after the surgery. One year postoperatively, the patient had pain-free full range of motion of the right knee without complaints. The Knee Society Score [22] was used to assess postoperative functional outcomes comparing them to the preoperative condition. The results showed improvement in pain and walking distance scores postoperatively.

## 4 Discussion

Intraarticular ganglion cysts of the knee joint are rare, with a reported prevalence of 0.2–1.3 % on MRI and 0.6 % during knee arthroscopy [4,6]. These cysts are benign lesions, and are extremely rare in pediatric age groups with fewer than ten cases reported in the literature [8,9,11,[13], [14], [15], [16], [17], [18]]. We report this case as the first infrapatellar fat pad ganglion cyst with anterolateral extraarticular extension via the lateral reticulum, compared to previous reported cases, which were pure intraarticular cysts the majority of which had an ACL origin.

Ganglion cysts are most commonly discovered incidentally during MRI or arthroscopy and approximately 90 % of the cases are asymptomatic [4]. However, knee pain was the most common complaint among the reported pediatric cases. Other shared symptoms are knee swelling; limping; and limited range of motion of the knee, either on extension or flexion in ACL and PCL ganglion cysts, respectively [9,[13], [14], [15], [16], [17], [18], [19]]. Regarding our case, the main complaint was an anterior knee mass associated with discomfort and limping. However, these symptoms are vague and inconclusive with regards to diagnosis, and further investigations were necessary like MRI.

Although intraarticular ganglion cysts are of unknown etiology, there are several theories including synovial herniation to the surrounding tissue, embryological displacement of the synovial tissue, trauma-induced degenerative changes, and mesenchymal pluripotent cell proliferation [6,14,16]. Regardless of the etiology, it is still difficult to diagnose. The presentation of similar clinical symptoms in pediatric patients should raise suspicion of a ganglion cyst. Since Liu et al. [12] suggested the possibility of a congenital etiology rather than induction by trauma or other mechanisms. Cysts are not strictly associated with specific clinical symptoms or a previous trauma. The clinical manifestation of a ganglion cyst in the knee is variable. Therefore, we advise clinicians to perform a radiographic work up to aid diagnosis in such cases.

In most of the reported cases, the cyst was either entirely intraarticular or extraarticular; surgical excision was performed by arthroscopy or open incision, respectively [6,[15], [16], [17],^20^]. However, the presentation was unique in our case, as the patient's cyst was intraarticular with extraarticular extension. We therefore opted to perform an arthrosporic removal of the intraarticular portion of the cyst via an arthroscopic portal incision and then utilized the same incision to extract the extraarticular extension of the cyst. The mass was completely detached, and the patient had a functioning distal nerve postoperatively.

Notably, in cases in which arthroscopic removal was used to extract the cyst, a lower risk of recurrence was observed than in cases wherein the cyst was removed by ultrasound or computed tomography-guided aspiration which show recurrence rate up to 60 % while recurrence rate around 9 % if managed by excision [4,16]. Despite the method of extraction, we believe that the total excision of the cyst while avoiding its rupture and leakage is the gold standard treatment. Such cysts must be completely removed while protecting the surrounding neurovascular structures. The work in this case report has been reported in line with the SCARE criteria [21].

In conclusion, we present herein a rare case of an unusual presentation and location of an intraarticular ganglion cyst in the knee joint of an otherwise healthy 5-year-old child. The cyst was extracted surgically by knee arthroscopy without any recurrence or complications. A very limited number of similar cases has been reported in the literature and none of them, to our knowledge, had extraarticular extension. This report aimed to increase awareness of the potentially unusual presentation of ganglion cysts and highlight the importance of using radiological investigation to obtain a diagnosis and plan appropriate surgical treatment.

The following is the supplementary data related to this article.

Video 1 Mass protrusion under the skin during knee flexion upon clinical examination. Video 1

## Author contribution

‐ Abdulmajeed Alwayil, MD, orthopedic surgeon, designed the manuscript, and wrote the manuscript.
‐ Raheef Alatassi, MD, MSc, orthopedic surgeon, performed the literature review, the data collection and wrote the manuscript.
‐ Aiman Alshomrani, MD, orthopedic surgeon, contributed to the manuscript writing.
‐ Khalid Beidas, MD, orthopedic surgeon, contributed to the manuscript writing.

## Consent

Written informed consent was obtained from the patient's parents for publication and any accompanying images. A copy of the written consent is available for review by the Editor-in-Chief of this journal on request.

## Ethical approval

Ethical approval for this study (SFHP-IRB research number: 24-741-38) was provided by Institutional Review Board, Riyadh, KSA on 12 September 2024.

## Guarantor

Dr. Abdulmajeed Alwayil, MD, SB-ORTHO, MBA.

## Research registration number

Not applicable.

## Funding

No specific grant from funding agencies in the public, commercial, or not-for-profit sectors was received for this work.

## Conflict of interest statement

The authors have no conflicts of interest to declare.
